# Exploring interaction with environmental affordances in schizophrenia spectrum disorders using virtual reality

**DOI:** 10.1038/s41537-026-00774-7

**Published:** 2026-06-25

**Authors:** Marco Kramer, Dustin Hirsch, Alice Sader, Julien Willms, Georg Juckel, Paraskevi Mavrogiorgou

**Affiliations:** Department of Psychiatry, LWL University Hospital, Ruhr University Bochum, Bochum, Germany

**Keywords:** Schizophrenia, Human behaviour

## Abstract

Schizophrenia has been associated with disturbances in perceiving affordances, i.e. action possibilities offered by the environment. However, empirical research has largely relied on static laboratory tasks that poorly capture the dynamic perception-action loops of everyday environments. In this exploratory study, we used immersive virtual reality (VR) to examine environmental exploration and interaction in patients with schizophrenia and healthy controls (*n* = 19 each). Participants completed baseline questionnaires including self-report measures of anomalous world experience and were exposed to natural and urban 360° video environments and an interactive VR game. Their field of view was recorded and manually coded using a predefined coding scheme. Subjective affect, stress, and presence were assessed before and after VR exposure. Patients with schizophrenia showed reduced visual exploration of the environments, reflected by fewer gaze shifts per minute compared with controls, particularly in urban scenes. In the interactive VR game, overall object interaction patterns were comparable, although patients showed a tendency toward longer latencies before initiating social interaction with a virtual non-player character. Reduced fixation of the character’s face was strongly associated with domains of anomalous world experience related to other persons, language, and atmosphere. Qualitative observations further suggested that immersive environments could interact with the sense-making of psychotic experiences in a few participants. Although preliminary given the limited sample size, these findings indicate subtle alterations in how patients with schizophrenia explore their surroundings, while basic object interaction remains preserved. Immersive VR paradigms may provide a promising experimental platform to investigate altered subject-world relations in psychosis.

## Introduction

Schizophrenia is a severe mental disorder associated with profound disability and reduced life expectancy^1,2^. Despite decades of extensive neurobiological research, its clinical impact has remained limited. In particular, the overall prognosis of schizophrenia has not improved, and new therapeutic approaches remain scarce^3,4^. These limitations have prompted calls for broader frameworks that complement neurobiological explanations with approaches addressing lived experience, embodiment, and the patient’s relation to the surrounding lifeworld^5^.

Phenomenological psychopathology has long emphasized that schizophrenia involves alterations in the person’s relation to a shared lifeworld rather than merely disturbances of isolated mental contents^6,7^. A systematic attempt to capture such disturbances of subject-world interaction is the Examination of Anomalous World Experience (EAWE), a phenomenologically informed interview assessing alterations across domains such as space and objects, time and events, other persons, language, atmosphere, and existential orientation^8,9^. Although conceptually influential, the EAWE has so far been applied in relatively few and small empirical studies, partly due to its methodologically demanding qualitative format. Previous studies identified characteristic patterns in schizophrenia, including destabilization of the experienced world and subjectivization, defined as “the dominance of one’s internal, subjective experiences in the perception or interpretation of the lived world”^10^. However, studies also highlighted considerable heterogeneity in how such world anomalies manifest across individuals^10–12^. The EAWE framework has also been shown to allow cultural and linguistic adaptation^13,14^.

From an ecological perspective, self-world relations can be described in terms of affordances, that is, possibilities for action offered by the environment relative to the capacities and concerns of the perceiver^15,16^. Affordances emerge within circular perception-action processes linking organism and environment. Perception is therefore not merely the passive registration of stimuli but an active orientation toward actionable possibilities, for example a surface appearing as walkable, an object as graspable, or another person as approachable or threatening. Importantly, these possibilities are not only motor opportunities but also reflect the perceiver’s concerns, values, and social roles^17^. Alterations in the field of relevant affordances have been proposed to characterize psychiatric disorders. For example, paranoid patients may experience the world as more threatening, whereas depressed patients may experience it as indifferent^16,17^. Recent work suggests that disturbances of the ecological self may lie at the core of schizophrenia, reflecting an impaired capacity to perceive affordances^18^. Such impairments may undermine meaningful engagement with the environment and manifest in disorganized behavior or a loss of the effortless, habitual interaction that normally characterizes everyday action^18,19^.

Empirical work partly supports this account. Patients with schizophrenia have been shown to be less accurate and slower in identifying the action possibilities of objects despite largely preserved perception of basic physical properties^20^, and to be less influenced by contextually congruent cues^19^. However, most studies have relied on simplified laboratory tasks involving explicit judgments of static objects presented on two-dimensional screens. Such paradigms capture only a narrow aspect of affordance perception and remain far removed from the dynamic perception-action loops of everyday environments. Methods that allow the ecologically valid observation of behavior in immersive and interactive settings are therefore needed^21^.

Immersive virtual reality (VR) offers a promising approach to address these limitations. Its use has increased substantially in recent years with the growing accessibility of head-mounted displays. In schizophrenia research, VR has already been used therapeutically, for example to target social cognition^22,23^, neurocognitive symptoms^24^, movement abnormalities^25^, or auditory verbal hallucinations^26,27^. At the same time, VR provides a powerful experimental tool to study how patients interact with their surrounding world^28^. It permits the systematic observation and manipulation of subject-world interactions, including spatial, temporal, and atmospheric properties of environments, and can thus function as a “phenomenological sandbox”. By enabling participants to move and act within complex three-dimensional environments, VR allows the investigation of perception-action processes under controlled yet ecologically richer conditions than traditional laboratory paradigms. Ethical considerations remain important, as the psychological consequences of immersive VR exposure are still insufficiently understood even in healthy populations^29^, and particularly in vulnerable groups such as individuals with schizophrenia.

Against this background, the present pilot study explored the emotional impact of immersive VR environments in patients with schizophrenia and their interaction with environmental affordances compared with healthy controls. To capture two complementary aspects of affordance-related subject-world-interaction, we implemented two conditions: In the first condition, we investigated the exploratory perception of affordances in 360° video environments by analyzing the visual focus on salient elements across different categories (e.g., moving/stationary objects, urban/natural scenes). We expected schizophrenia spectrum disorders to be associated with reduced or altered environmental exploration. In a second condition, we were specifically interested in the controller-based interaction with virtual objects and a social non-player character, to thereby probe the overt interaction with enacted object-related and social affordances. We expected schizophrenia spectrum disorders to be associated alterations in interacting with environmental affordances, especially in socially salient interactions. In addition, we explored whether behavioral patterns observed in VR environments were associated with domains of anomalous world experience derived from the EAWE framework. By combining behavioral observation with phenomenologically informed measures of subject-world experience, the study aimed to experimentally probe how alterations of the lived world in schizophrenia manifest in real-time perception-action dynamics.

## Materials and methods

### Participants

Participants comprised patients with schizophrenia spectrum disorders and healthy control subjects aged 18–70 years. Patients were recruited from their in- or outpatient treatment at the LWL-University Hospital of the Ruhr-University Bochum between September 2022 and February 2024, whereas healthy controls were recruited from the local community through advertisement during the same period.

Patients were eligible if they had a schizophrenia spectrum disorder (ICD-10 F2x.x) diagnosed by an experienced psychiatrist as part of routine clinical care. Additional inclusion criteria were sufficient German language proficiency, capacity to provide informed consent, and self-reported abstinence from illicit drug use for at least 6 months prior to participation. Healthy controls were required to have no history of psychiatric or neurological disorders and not to be taking psychotropic medication.

Exclusion criteria for both groups included alcohol dependence (ICD-10 F10.2), recent illicit drug use, severe personality disorders, organic brain disorders, acute suicidality or aggressive behavior, and other conditions preventing safe participation in the VR experiment. Patients with an acute and insufficiently treated psychotic episode were not included. Pregnant or breastfeeding women were excluded.

The study was planned as an exploratory pilot study. Sample size estimation was discussed in close consultation with biostatisticians from the Department of Medical Informatics, Biometry and Epidemiology at Ruhr University Bochum. Due to the heterogeneous previous literature and the exploratory nature of the study, an a priori power calculation was not feasible. The target sample size was therefore heuristically determined as approximately 20 participants with schizophrenia spectrum disorders and 20 healthy controls, which was considered appropriate for an exploratory pilot study. A total of 20 patients with schizophrenia and 20 healthy controls were initially recruited. One control participant discontinued the experiment, and one patient withdrew consent for personal reasons and requested deletion of all data. The final sample, therefore, consisted of 19 patients with schizophrenia and 19 healthy controls.

A sensitivity power analysis conducted in G*Power 3.1.9.7^30^ indicated that, with *n* = 19 participants per group, *α* = 0.05 and 80% power, the study was sufficiently powered to detect only large between-group effects of approximately Cohen’s *d* = 0.93. For exploratory correlation analyses within the patient group, the corresponding detectable effect size was approximately *r* = 0.53. Thus, the study was underpowered to detect small or moderate effects, particularly after correction for multiple comparisons.

The study, including its planned sample size, was approved by the local ethics committee of the Medical Faculty of the Ruhr-University-Bochum (reference number: 22-7575), and all participants provided written informed consent prior to participation. Each participant received €20 as compensation.

### Study design

**Fig. 1:**
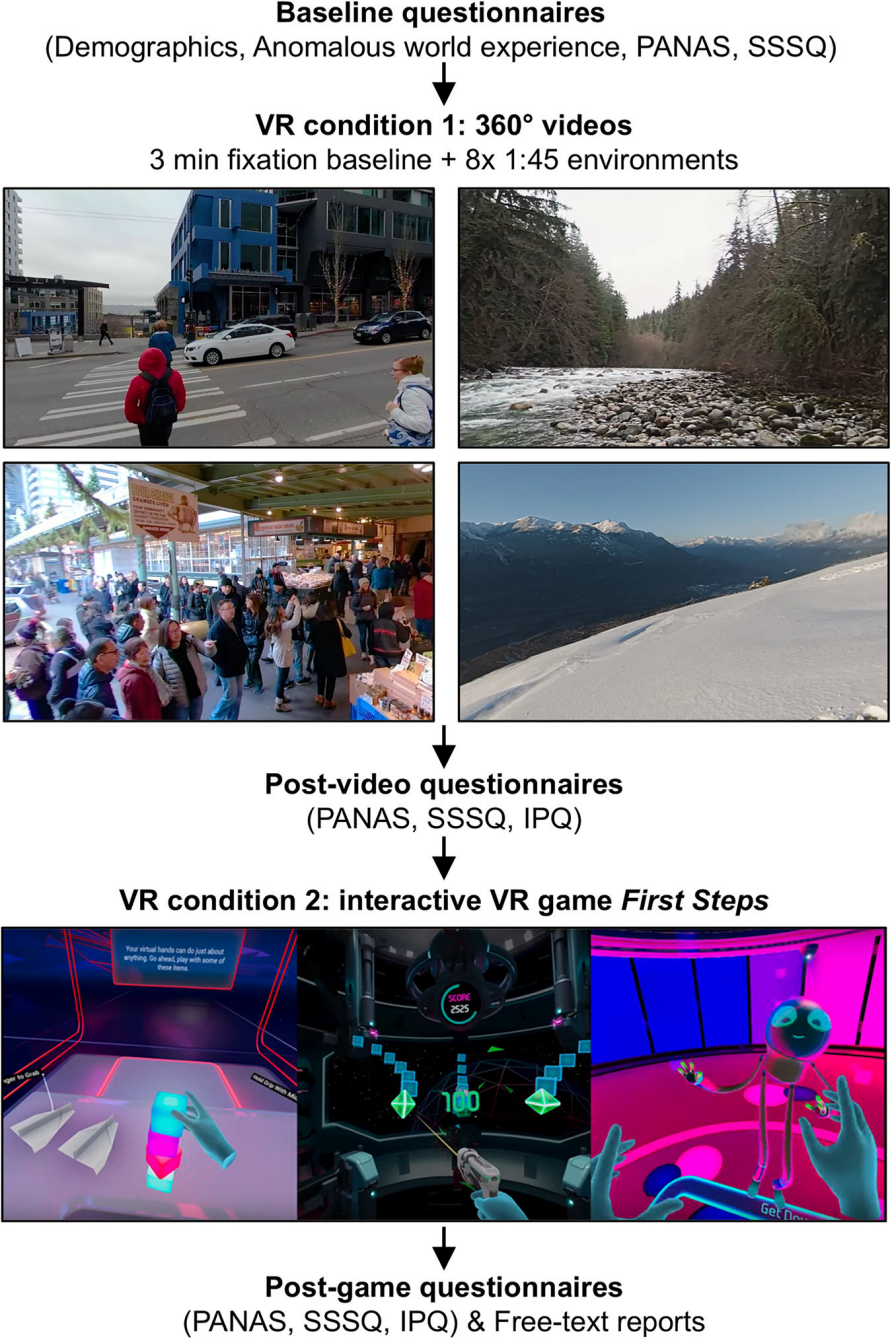
Experimental procedure. EAWE examination of anomalous world experience, PANAS positive and negative affect schedule, SSSQ short stress state questionnaire, IPQ iGroup presence questionnaire. Representative 360° video screenshots reproduced with permission for illustrative purposes by ProArtInc (www.proartwa.com) and screenshots from “First Steps” reproduced with permission by Meta Platforms, Inc.

The experimental procedure is illustrated in Fig. 1. After providing informed consent, participants first completed baseline questionnaires assessing demographic characteristics, anomalous world experience (EAWE-derived self-report), affect (PANAS), and stress (SSSQ).

Before each experimental condition, a 3-min baseline video consisting of a black background with a white fixation cross was presented to establish a baseline level of physiological arousal. During the baseline and all subsequent VR exposures, participants stood upright and were allowed to move freely within a 2 × 2 m^2^ area.

Participants were then exposed to two VR conditions presented using a Meta Quest 2 headset (Meta Platforms Inc., Menlo Park, CA, USA). In the first condition, participants viewed a sequence of 360° videos. In the second condition, participants engaged in an interactive VR game.

The order of conditions was fixed (videos followed by the interactive task), and not counterbalanced, to allow participants, most of whom had limited prior VR experience, to become familiar with VR in a task-free setting before the cognitive and sensorimotor demands increased during controller-based interaction. In addition, presenting the interactive task first might have created expectations about action possibilities and thereby contaminated spontaneous viewing behavior in subsequent 360° videos.

The duration of the interactive game condition was self-paced, allowing participants to explore the virtual environment until they considered the available interaction possibilities sufficiently explored. This design was chosen to preserve the exploratory character of the task and to avoid hindering participants from interacting with all available objects. However, because self-paced exposure may introduce variability in behavioral measures, total task duration was documented and is reported in the Results section.

During VR exposure, the participants’ field of view was mirrored to a desktop computer and recorded using the open-source software Open Broadcaster Software Studio (OBS Studio; OBS Project, San Francisco, CA, USA) at a resolution of 1280 × 720 pixels and 30 fps for further analysis.

After each VR condition, participants completed questionnaires assessing affect (PANAS), stress (SSSQ), and presence (IPQ). Finally, participants provided open-ended written and oral reports describing their subjective experiences during the VR exposure.

### Virtual environments

#### 360° video environments

The first experimental condition consisted of a 360° video sequence comprising eight video segments depicting natural environments (beach, desert, mountaintop, savanna, forest, riverside) and urban environments (street and urban market). Each segment lasted 1 min 45 s. The videos were sourced from publicly available 360° content on YouTube (YouTube, LLC, San Bruno, CA, USA). The original audio tracks were played identically to all participants and consisted exclusively of environment-typical ambient sounds without music or narration. A complete list of video sources and creators is provided in Supplementary Table 1.

Participants could freely explore the scenes through head and body movements but did not use handheld controllers during the video condition.

#### Interactive VR environment

Interactive behavior was assessed using the VR tutorial application *First Steps* (Oculus Studios, Meta Platforms, Menlo Park, CA, USA), which is freely available for Meta Quest devices. The application is designed as an introductory experience demonstrating controller-based interaction with virtual objects.

Participants first entered a futuristic lobby environment in which they learned to control virtual hands using handheld controllers and interact with several objects. Objects were introduced sequentially and included graspable toy blocks, a throwable paper plane, a remote-controlled toy airship, a small punching bag, a toy rocket with a pullable label, and a table-tennis bat with ball.

After this sequence, two additional scenarios could be accessed. One consisted of a shooting-gallery-like environment in which abstract objects could be targeted using different virtual weapons. The other consisted of a dance interaction with a friendly robot-like non-player character (NPC), which invited participants to dance, and which mirrored the participants’ movements. During the dance interaction, short text prompts encouraged participants to perform different movements (e.g., “Move your hands!” or “Grab a hand!”).

Because the present study focused on exploratory behavior and social interaction, analyses were restricted to the lobby and dance scenarios. The shooting-gallery environment was not included in the analysis.

### Measures

#### Psychometric measures

Affect was assessed using the validated German version of the Positive and Negative Affect Schedule (PANAS)^31^ adapted from the original English version^32^. The instrument consists of two scales measuring positive and negative affect, with items rated on a 5-point Likert scale (1 = “not at all” to 5 = “extremely”).

Stress was measured using the Short Stress State Questionnaire (SSSQ), which comprises 24 items across three subscales (engagement, distress, worry), each rated on a 5-point Likert scale (1 = “not at all” to 5 = “completely”)^33^.

Presence during the VR conditions was assessed using the iGroup Presence Questionnaire (IPQ)^34^. The instrument comprises 14 items rated on 7-point Likert scales and includes three subscales (spatial presence, involvement, experienced realism) as well as one general presence item.

Psychotic symptom severity in the patient group was assessed using the Positive and Negative Syndrome Scale (PANSS), which employs a 7-point rating scale (1 = “not present” to 7 = “extreme”) and yields scores for positive symptoms, negative symptoms, and general psychopathology^35^.

Anomalous world experience was assessed using a self-developed self-report questionnaire conceptually derived from the German version of the Examination of Anomalous World Experience (EAWE)^8,9^. The instrument comprised 47 items rated on a 5-point Likert scale (1 = “never” to 5 = “all the time”) and assessed alterations in the domains Space and Objects, Time and Events, Other Persons, Language, Atmosphere, and Existential Orientation.

#### Visual exploration in 360° videos

Visual exploration of the virtual environments was quantified based on recordings of the participants’ field of view during VR exposure (see “Study design”).

Following an initial inspection of the recordings, the study team (D.H. and M.K.) discussed salient elements within the video environments, i.e., visually prominent and/or behaviorally meaningful scene components, that are frequently looked at by participants independently of group membership and could plausibly structure participants’ orientation within the environment. The study team then agreed on a coding manual defining salient visual elements. For example, the following codes were designed for the urban environment in the tile at the top left of Fig. 1: Scene with people in the foreground vs. scene without people in the foreground; focus on the street layout, sky/skyscrapers, crosswalk, intersection, and/or “person X” (a specific salient person waiting at the traffic light next to the 360° camera). Based on this coding scheme, time segments were manually coded in which a predefined salient object or scene element was in the central field of view of the participant by one investigator (D.H.) using MAXQDA Analytics 26.1.0 (VERBI Software, Berlin, Germany), who was blinded to group membership. These coded segments were used to derive measures of visual exploration. The number of transitions between different salient elements was used to calculate gaze shifts per minute as an index of exploratory behavior.

#### Behavioral interaction in the VR game

Behavioral interaction during the interactive VR game was quantified using similar video recordings of the participants’ field of view.

Again, a coding manual was developed by the study team (D.H. and M.K.) after visual inspection of the material. Since there were no established VR paradigms on which this pilot study could build, the investigated codes were partly inspired by previous research on affordances focusing on reaction times/latencies and action types^19,20^, partly grounded in the assumed affordances offered by the employed virtual environments and partly based on actual interactions seen in the material. The following interaction metrics were manually coded by one investigator (D.H.) using MAXQDA Analytics 26.1.0 (VERBI Software, Berlin, Germany), who was blinded to group membership: First, difficulties controlling the virtual hands were assessed throughout the interactive task. These were operationalized as repeated unsuccessful controller-based attempts to perform an apparently intended interaction with a virtual object before the action was successfully executed.

Furthermore, reaction times to newly appearing objects, types of interactions with objects, and interaction durations were coded for the lobby sequence.

For the dance interaction with the non-player character (NPC), additional measures were derived. These included the latency until initiation of the dance interaction, face fixation toward the NPC, defined as the robot’s face appearing in the central field of view, and touch interaction (i.e., grasping the robot’s virtual hands).

### Subjective experience reports

Participants were asked to describe their subjective experiences during the VR exposure in open-ended written reports. Responses were documented either by the participants themselves or by study personnel and were analyzed descriptively due to their brief and heterogeneous nature.

### Statistical analysis

Statistical analyses were performed using IBM SPSS Statistics 29.0.0.1 (IBM Corp., Armonk, NY, USA). Continuous variables are reported as mean ± standard deviation (SD) unless otherwise indicated.

Group differences in demographic and baseline variables were assessed using independent-samples t-tests, Mann–Whitney U tests, or *χ*² tests, depending on measurement scale and distributional properties.

To examine group differences in visual exploration and behavioral interaction measures, analyses of covariance (ANCOVAs) were conducted with group as the between-subject factor and age as a covariate. For variables measured across stimulus categories (e.g., environment type or object category), repeated-measures ANCOVAs were performed with the respective category as the within-subject factor. Group differences regarding action types were assessed by use of two-sided Fisher’s exact tests. Effect sizes are reported as partial eta squared (pη²).

Associations between behavioral measures, psychopathology (PANSS), and anomalous world experience (EAWE-derived domains) were examined using Spearman rank correlations, given the relatively small sample size and non-normal distribution of several variables.

To assess medication effects on main outcome variables, chlorpromazine equivalents based on the patients’ medications were calculated using the R package *chlorpromazineR 0.2.0* in RStudio 2026.01.0 (Posit Software, PBC, Boston, MA) with *leucht2020* as primary conversion key^36^ and, in one case of long-acting injectable haloperidol by *leucht2016* as conversion key^37^. Equivalent doses were summed per participant and used for exploratory correlation analyses using Spearman rank correlations.

Unless otherwise indicated, p-values were adjusted using the Benjamini–Hochberg false discovery rate (FDR) procedure to control for multiple comparisons^38^. All tests were two-tailed, and statistical significance was defined as *p* < 0.05. Results that did not survive correction for multiple comparisons are reported as uncorrected exploratory findings and should be interpreted with caution.

## Results

### Demographic and psychometric results

An overview of demographic and psychometric results is shown in Table 1. Participants with schizophrenia spectrum disorders were significantly older than controls but did not differ in sex distribution. Age was therefore included as a covariate in subsequent analyses.

**Table 1:** Demographic and psychometric results.

Variable	SCZ (*n* = 19)	HC (*n* = 19)	Test statistics	
Demographics				
- Sex (*n*, % female) - Age	6, 31.58% 38.95 ± 8.80	11, 57.89% 29.11 ± 8.10	*χ*^2^(1) = 2.661, *p* = 0.191, *φ* = 0.27 *U* = 62.5, *Z* = −3.45, *p* < 0.001***	
Baseline psychometry				Age-adjusted ANCOVA:
- Negative affect (PANAS) - Positive affect (PANAS) - Engagement (SSSQ) - Distress (SSSQ) - Worry (SSSQ)	1.46 ± 0.61 3.47 ± 0.79 28.84 ± 12.72 13.44 ± 6.11 20.89 ± 6.63	1.44 ± 0.49 3.39 ± 0.68 28.21 ± 3.07 9.95 ± 2.15 20.11 ± 5.23	*U* = 180.0, *Z* = 0.276, *p* = 0.799 *U* = 153.0, *Z* = −0.549, *p* = 0.799 *U* = 152.5, *Z* = −0.820, *p* = 0.627 *U* = 109.0, *Z* = −1.91, *p* = 0.183 *t*(35) = 0.400, *p* = 0.784	*F*(1,34) = 0.11, *p* = 0.744, *pη*² = 0.01 *F*(1,34) = 0.50, *p* = 0.744, *pη*² = 0.02 *F*(1,35) = 0.12, *p* = 0.733, *pη*² = 0.01 *F*(1,35) = 1.07, *p* = 0.509, *pη*² = 0.03 *F*(1,34) = 0.94, *p* = 0.509, *pη*² = 0.03
Baseline EAWE-derived scores				
- Space and objects - Time and events - Other persons - Language - Atmosphere - Existential orientation	1.87 ± 0.84 1.98 ± 1.06 2.40 ± 0.94 2.13 ± 0.68 1.91 ± 0.72 2.67 ± 0.76	1.23 ± 0.45 1.21 ± 0.54 1.81 ± 0.73 1.57 ± 0.73 1.19 ± 0.52 1.87 ± 0.68	*U* = 87.0, *Z* = −2.74, *p* = 0.012* *U* = 114.0, *Z* = −1.97, *p* = 0.053 *U* = 114.0, *Z* = −1.95, *p* = 0.053 *U* = 101.5, *Z* = −2.32, *p* = 0.030* *U* = 72.0, *Z* = −3.20, *p* = 0.006** *t*(36) = 3.421, *p* = 0.006**	*F*(1,35) = 5.49, *p* = 0.025*, *pη*² = 0.14 *F*(1,35) = 6.52, *p* = 0.024*, *pη*² = 0.16 *F*(1,35) = 5.54, *p* = 0.025*, *pη*² = 0.14 *F*(1,35) = 6.42, *p* = 0.024*, *pη*² = 0.16 *F*(1,35) = 11.61, *p* = 0.012*, *pη*² = 0.25 *F*(1,35) = 7.21, *p* = 0.024*, *pη*² = 0.17
Post-video			Age-adjusted ANCOVAs:	
- Negative affect (ΔPANAS) - Positive affect (ΔPANAS) - Engagement (ΔSSSQ) - Distress (ΔSSSQ) - Worry (ΔSSSQ) - IPQ: General Presence - IPQ: Spatial Presence - IPQ: Involvement - IPQ: Realism	−0.06 ± 0.40 −0.06 ± 0.66 −0.47 ± 11.04 −1.61 ± 6.36 +1.22 ± 5.83 3.84 ± 2.54 19.79 ± 8.22 15.26 ± 5.86 12.21 ± 3.85	−0.08 ± 0.46 −0.27 ± 0.79 −2.95 ± 3.76 −0.11 ± 2.87 −1.21 ± 2.59 4.26 ± 1.56 18.89 ± 4.74 14.95 ± 3.55 13.37 ± 3.40	*F*(1,31) = 0.38, *p* = 0.540, *pη*² = 0.01 *F*(1,31) = 0.60, *p* = 0.540, *pη*² = 0.02 *F*(1,35) = 0.68, *p* = 0.653, *pη*² = 0.02 *F*(1,34) = 0.04, *p* = 0.852, *pη*² < 0.01 *F*(1,34) = 0.62, *p* = 0.653, *pη*² = 0.02 *F*(1,35) = 0.55, *p* = 0.789, *pη*² = 0.02 *F*(1,35) = 0.05, *p* = 0.823, *pη*² < 0.01 *F*(1,35) = 0.62, *p* = 0.789, *pη*² = 0.02 *F*(1,35) = 0.29, *p* = 0.789, *pη*² = 0.01	
Post-game				
- Negative affect (ΔPANAS) - Positive affect (ΔPANAS) - Engagement (ΔSSSQ) - Distress (ΔSSSQ) - Worry (ΔSSSQ) - IPQ: General Presence - IPQ: Spatial Presence - IPQ: Involvement - IPQ: Realism	−0.04 ± 0.29 +0.45 ± 0.77 +2.21 ± 4.71 −1.26 ± 3.36 +0.26 ± 2.62 4.84 ± 2.17 23.89 ± 5.83 18.68 ± 4.07 12.26 ± 4.25	−0.06 ± 0.36 +0.41 ± 0.72 +1.53 ± 4.78 −1.16 ± 1.95 −1.11 ± 3.40 4.95 ± 2.01 21.84 ± 5.33 14.95 ± 3.55 13.00 ± 2.56	*F*(1,32) = 0.51, *p* = 0.559, *pη*² = 0.02 *F*(1,32) = 0.35, *p* = 0.559, *pη*² = 0.01 F(1,35) = 0.38, *p* = 0.636, *pη*² = 0.01 *F*(1,35) = 0.23, *p* = 0.636, *pη*² = 0.01 *F*(1,35) = 2.04, *p* = 0.486, *pη*² = 0.06 *F*(1,35) = 0.07, *p* = 0.796, *pη*² < 0.01 *F*(1,35) = 0.10, *p* = 0.796, *pη*² < 0.01 *F*(1,35) = 4.48, *p* = 0.168, *pη*² = 0.11 *F*(1,35) = 0.50, *p* = 0.796, *pη*² = 0.01	

Δ refers to post-pre-differences. All values indicate mean ± SD, unless otherwise indicated. *p*-values have been corrected using the Benjamini-Hochberg procedure.

*SCZ* schizophrenia, *HC* healthy control, *PANAS* positive and negative affect schedule, *SSSQ* short stress state questionnaire, *EAWE* examination of anomalous world experience, *IPQ* iGroup presence questionnaire.

Prior experience with VR was reported by three patients (15.8%) and seven healthy controls (36.8%). Only one control participant reported regular VR use. Weekly duration of virtual media use did not differ significantly between groups.

The patient group included 17 participants with paranoid schizophrenia (F20.0), 1 with hebephrenic schizophrenia (F20.1), and 1 with schizoaffective disorder (F25.1). Most patients with schizophrenia spectrum disorders (94.7%) were receiving psychotropic medication. The majority were treated with second-generation antipsychotics (84.2%) while one patient received a first-generation antipsychotic and four received antidepressants (sertraline or bupropion). The mean PANSS total score was 52.72 ± 20.30.

At baseline, groups did not differ in subjective affect (PANAS) or stress (SSSQ). In the EAWE-based self-report questionnaire, patients with schizophrenia showed significantly higher scores than controls in the domains *Space and Objects, Language, Atmosphere*, and *Existential Orientation*, whereas in the subscales *Time and Events* and *Other persons* there was no significant difference after correction for multiple comparisons. When the baseline variables were adjusted for age using age-adjusted ANCOVAs, no significant differences were found regarding subjective affect (PANAS) or stress (SSSQ). However, in this age-adjusted analysis, significant group differences were found for all EAWE-based subscales, even after correction for multiple comparisons.

To assess exposure-related group differences, subjective presence ratings (IPQ) after exposure and post-pre differences in affect (PANAS) and stress (SSSQ) were compared between groups separately for the video and game conditions using age-adjusted ANCOVAs. No significant group differences were observed.

### Visual exploration of virtual environments

**Fig. 2:**
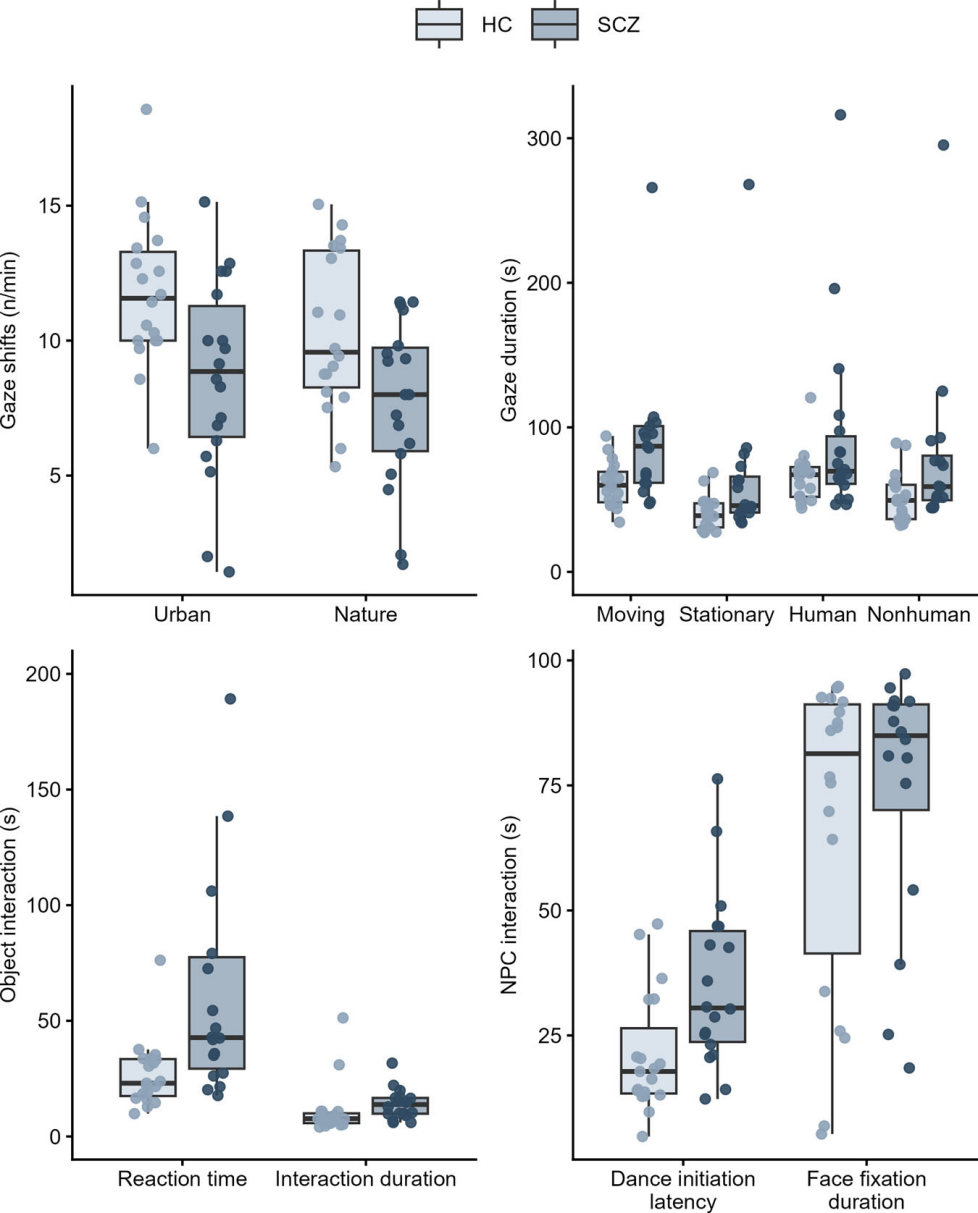
Group differences in visual exploration and behavioral interaction. Boxplots for patients with schizophrenia (SCZ) and healthy controls (HC). Boxes represent the interquartile range with the median indicated by the central line. Whiskers extend to 1.5×IQR. Points represent individual participants. * = corrected *p* < 0.05, † = uncorrected *p* < 0.05.

We investigated group differences in visual exploration of the virtual environments by analyzing transitions between salient scenes and objects as well as gaze durations for different object categories. Results were analyzed using age-adjusted repeated-measures ANCOVAs (Table 2). The upper panels of Fig. 2 present the results for gaze shifts and gaze durations.

**Table 2:** Behavioral variables in 360° videos and a virtual game.

Variable	SCZ (*n* = 19)	HC (*n* = 19)	Test statistics (age-adjusted)
360° videos: Gaze shifts [*n*/min]			
- Urban scene - Natural scene	8.62 ± 3.71 7.70 ± 3.05	11.43 ± 3.07 10.00 ± 3.18	*F*(1, 34) = 5.55, *p* = 0.048*, *pη*² = 0.14 *F*(1, 34) = 1.61, *p* = 0.213, *pη*² = 0.05
360° videos: Gaze duration [s]			
- Moving objects - Stationary objects - Other persons - Non-human objects	111.36 ± 99.98 66.38 ± 59.20 93.86 ± 66.79 80.17 ± 61.64	60.88 ± 15.49 40.70 ± 11.95 65.67 ± 17.64 51.13 ± 17.25	*F*(1, 32) = 2.61, *p* = 0.232, pη² = 0.08 *F*(1, 31) = 1.16, *p* = 0.289, *pη*² = 0.04 *F*(1, 33) = 1.29, *p* = 0.295, *pη*² = 0.04 *F*(1, 31) = 1.14, *p* = 0.295, *pη*² = 0.04
Virtual game: object interaction			
- Control difficulties [s] - Action diversity [n] - Reaction time [s] - Interaction duration [s]	62.58 ± 74.52 8.72 ± 2.56 64.79 ± 12.35 37.07 ± 7.62	24.12 ± 29.76 8.37 ± 2.65 37.90 ± 12.35 24.56 ± 7.39	*F*(1, 34) = 0.68, *p* = 0.432, pη² = 0.02 *F*(1, 34) = 0.63, *p* = 0.432, *pη*² = 0.02 *F*(1, 33) = 2.09, *p* = 0.432, *pη*² = 0.06 *F*(1, 34) = 1.22, *p* = 0.432, *pη*² = 0.04
Virtual game: NPC interaction [s]			
- Dance initiation latency - Face fixation duration - Touch interaction	35.57 ± 17.25 74.31 ± 25.52 27.69 ± 17.63	21.13 ± 11.89 66.58 ± 31.91 23.83 ± 17.29	*F*(1, 34) = 6.19, *p* = 0.054, pη² = 0.15 *F*(1, 31) = 0.17, *p* = 0.782, *pη*² = 0.01 *F*(1, 31) = 0.08, *p* = 0.782, *pη*² = 0.00

Statistics are presented as mean ± SD, effect sizes as partial eta square. *p*-values have been corrected using the Benjamini-Hochberg procedure.

*SCZ* schizophrenia, *HC* healthy control, *NPC* non-player character.

Overall, patients showed significantly fewer gaze shifts per minute than healthy controls (16.32 ± 6.35 vs. 22.06 ± 5.26, *F*(1, 33) = 4.995, *p* = 0.032, *pη*² = 0.13). When calculated separately for urban and natural scenes and averaged across the respective video segments, the group difference was evident for urban scenes with a large effect size but not for natural scenes.

Mean gaze durations were further analyzed for objects from specific classes (moving vs. stationary objects; human vs. non-human objects), averaged across all videos. Patients showed descriptively longer gaze durations across object categories; however, none of these differences remained significant after correction for multiple comparisons.

### Behavioral interaction with the virtual environment

We analyzed group differences in behavioral engagement with the virtual environment during the interactive VR game, including hand control, interaction with virtual objects, and interaction with a non-player character (NPC; Table 2). The lower panels of Fig. 2 present the results for object and NPC interactions.

An age-adjusted ANCOVA showed no group difference in the time required to learn to control the virtual hands, indicating comparable ability to embody the virtual hands and operate the interaction mechanics. However, age was associated with greater difficulty controlling the virtual hands (*F*(1,34) = 4.54, *p* = 0.040, *pη*² = 0.12).

In the lobby sequence, the mean duration of interaction with objects was 637.18 ± 506.67 s in patients and 313.61 ± 104.05 s in controls. An age-adjusted ANCOVA showed no group difference (*F*(1,34) = 1.92, *p* = 0.175, *pη*² = 0.05), but a significant influence of age on interaction duration (*F*(1,34) = 4.84, *p* = 0.035, *pη*² = 0.13). The distribution of action types was similar between groups (Supplementary Table 2). Within-individual action diversity, defined as the number of different interaction types performed across objects, also did not differ between groups. Mean reaction times to newly appearing objects and mean interaction durations were descriptively slower in patients. However, the age-adjusted group difference was not significant. Age showed a significant effect on reaction times (*F*(1,33) = 4.70, *p* = 0.037, *pη*² = 0.13), whereas neither the main effect of object nor the group x object interaction reached significance. Interaction durations showed no significant group, group x object or age effect.

In the dance sequence, the mean duration of interaction with the NPC was 180.79 ± 93.94 s in patients and 151.91 ± 56.87 s in controls. An age-adjusted ANCOVA showed no group difference *(F*(1,34) = 0.09, *p* = 0.771, *pη*² = 0.003), but a significant influence of age on interaction duration (*F*(1,34) = 7.45, *p* = 0.010, *pη*² = 0.18). In the dance sequence, three patients with schizophrenia spectrum disorders (15.8%) did not initiate dancing with the NPC, whereas all healthy controls did. Among participants who initiated dancing, patients showed longer latencies before dance initiation with a large effect size. After correction for multiple comparisons using the FDR, this difference was no longer significant (corrected *p* = 0.054). Durations of face fixation and touch interaction with the NPC did not differ between groups.

### Associations with psychopathology, medication, and anomalous world experience

**Fig. 3:**
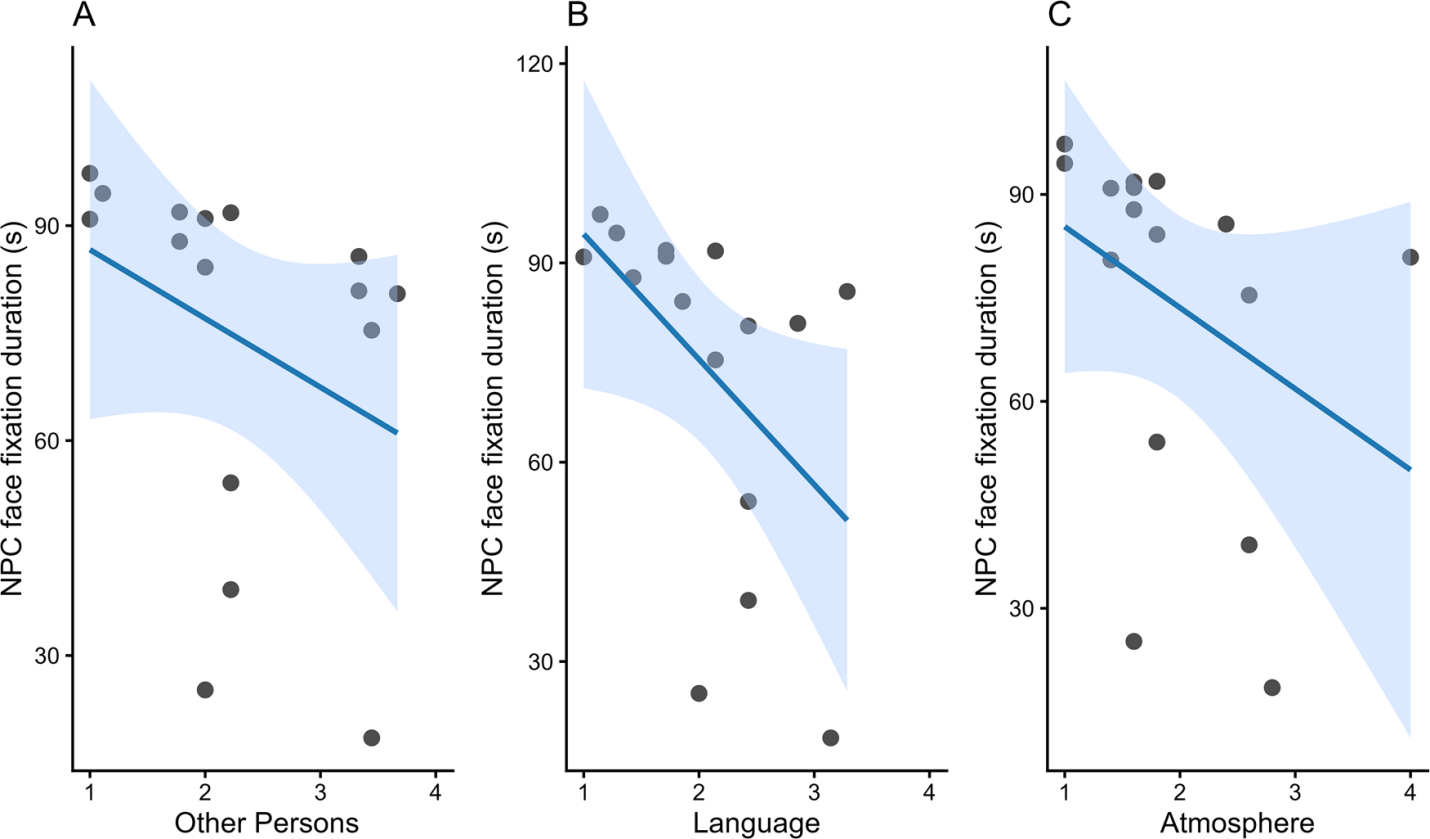
Correlations between EAWE-derived domains and NPC face fixation. Scatterplots showing associations between EAWE-derived domain scores (**A**, other persons; **B**, language; **C**, atmosphere) and face fixation duration toward a virtual non-player character (NPC) in patients with schizophrenia. Points represent individual participants. Lines (blue) indicate linear regression with 95% confidence intervals (light blue).

We explored whether measures of interaction with the virtual environments were associated with symptom severity and domains of anomalous world experience (EAWE; Supplementary Table 3; Fig. 3).

Strong negative correlations were observed between NPC face fixation duration and the EAWE domains *Other Persons* (*r* = −0.669, corrected *p* = 0.018), *Language* (*r* = −0.677, corrected *p* = 0.018), and *Atmosphere* (*r* = −0.615, corrected *p* = 0.018; Fig. 3). PANSS scores and other EAWE-derived domains did not show comparable relationships.

The PANSS total score was positively associated with difficulties controlling virtual objects during the game sequence (*r* = 0.660, *p* = 0.028), in contrast to EAWE-derived domain scores.

Several additional associations did not survive correction for multiple comparisons and are therefore reported as exploratory. The EAWE domain *Time and Events* showed a moderate association with the PANSS total score (*r* = 0.588, raw *p* = 0.01, corrected *p* = 0.06). The domain *Other Persons* showed a moderate association with longer gaze durations for non-human objects (*r* = 0.456, raw *p* = 0.049, corrected *p* = 0.319). The domain *Language* was moderately negatively associated with mean interaction duration in the VR game (*r* = −0.509, raw *p* = 0.031, corrected *p* = 0.203).

Medication effects were explored by exploratory correlations with chlorpromazine equivalents (Supplementary Table 4). Exploratory Spearman correlations showed no FDR-corrected associations between chlorpromazine-equivalent daily dose and PANSS total score or the main VR outcome variables. Chlorpromazine-equivalent dose showed a non-significant positive association with PANSS total score (*r* = 0.477, raw *p* = 0.061, corrected *p* = 0.366). Among the behavioral variables, only object interaction duration showed an uncorrected negative association with chlorpromazine-equivalent dose (*r* = −0.553, raw *p* = 0.026), which did not survive correction (corrected *p* = 0.324).

### Descriptive observations of subjective experiences

Subjective experiences in the virtual environments were systematically collected and documented by participants or study personnel and are summarized descriptively due to their heterogeneous nature. Labels named “VPP” refer to participant identifiers from the patient group.

Most patients reported neutral or positive experiences in the virtual environments. Several described the VR game as fun (VPP4, VPP6, VPP16), impressive (VPP8), restorative (VPP14), or distracting from negative thoughts (VPP16). Others reported increased curiosity (VPP15) or feelings of self-efficacy (VPP9, VPP12). A minority reported ambivalent or negative experiences, such as boredom during the videos (VPP4, VPP12) or transient stress reactions (VPP16).

Three patients explicitly linked aspects of the VR experience to their psychotic experiences. One participant reported briefly perceiving “someone who wasn’t there”, which was experienced as stressful (VPP16). Another described deliberately interacting with virtual objects in an attempt to recreate a perceptual experience from a previous psychotic episode, in which objects had appeared to move toward each other (VPP2). A third participant with a persistent delusional belief involving a parasitical alien reported that the VR experience enabled her or him to meet the perceived entity “on the same plane” for the first time and made “the boundary between me and my alien friend thinner” (VPP6). This participant described this as the beginning of a dialogic interaction with the perceived entity, for which follow-up consultation was sought.

These reports suggest that in a minority of patients, prior psychotic experiences could shape how participants interpreted action possibilities in virtual environments or influence the sense-making of ongoing psychotic experiences.

## Discussion

The present study suggests there might be subtle alterations in how patients with schizophrenia spectrum disorders visually explore and interact with immersive virtual environments. Moreover, specific interaction patterns were associated with domains of anomalous world experience. Taken together, these findings suggest that VR may provide a promising experimental window into altered subject-world relations in psychosis.

### Altered interaction with environmental affordances

Patients with schizophrenia spectrum disorders showed reduced visual exploration of the virtual environments, as reflected by fewer gaze shifts between salient objects and scenes. This pattern suggests a reduced sampling of environmental information and a more prolonged engagement with individual elements of the scene.

Altered visual exploration has been consistently reported in schizophrenia^39^. Eye-tracking studies show that patients produce fewer fixations when viewing complex images or movies, are less driven by bottom-up saliency and thus focus later on salient image regions, while exhibiting reduced neural responses associated with visual attention^40,41^. On a brain network level, such impairments of salience-guided eye movements were also related to abnormalities in the magnocellular visual pathway^42^.

The present findings extend this literature to immersive VR environments. Notably, the effect was mainly observed during the passive 360° video condition and was more pronounced in urban than natural scenes. This is of interest given that urban environments represent a well-established risk factor for schizophrenia^43^ and qualitative studies suggest that psychotic patients may relate differently to urban environments^44^. However, research based on patients’ experience of and interaction with the urban environment is scarce^45^. Given the small sample size, these findings should not be overinterpreted but warrant further research.

Importantly, reduced exploration did not translate into clear impairments in object interaction during the VR game. Face fixation during the dance interaction with the virtual character and action durations were largely comparable between groups, suggesting that alterations may primarily concern exploratory sampling of the environment.

Furthermore, participants with schizophrenia spectrum disorders were generally able to learn the basic interaction mechanics of the VR environment and control the virtual hands, indicating that the sensorimotor coupling required for embodied interaction remained largely intact. At the same time, patients showed descriptively slower reactions to newly appearing objects and longer latencies before initiating social interaction with the non-player character. However, these effects did not remain significant after correction for age and multiple comparisons, so they may be attributable to age rather than schizophrenia spectrum disorder per se. Future studies in larger, better age-matched samples may explore to what extent these effects might also be shaped by subtle alterations in the transition from perceiving to acting upon environmental opportunities in schizophrenia.

Finally, immersive VR provides a methodological advantage over traditional eye-tracking paradigms. Conventional eye-tracking studies typically present static or pre-recorded stimuli in passive viewing situations. By contrast, ecological approaches to perception emphasize that perception is inherently action-oriented and embedded within a field of affordances. Immersive VR environments allow participants to move, explore, and act within such fields of possibilities while maintaining experimental control over environmental features. In this sense, VR can be conceptualized as a “phenomenological sandbox” that enables the systematic investigation of subject-world relations under controlled yet ecologically rich conditions. Although preliminary in nature, the present pattern of reduced exploration but largely preserved interaction once objects are engaged may therefore suggest that the disturbance lies less in the motor execution of actions than in how potential actions become salient and are detected within the surrounding field of affordances. Future studies with larger samples should examine whether such alterations are particularly pronounced in urban contexts, either by applying eye-tracking in natural environments or by employing interactive VR paradigms.

### Association with anomalous world experience

In the present study, reduced fixation on the NPC’s face was consistently associated with higher scores in the EAWE domains *Other Persons*, *Language*, and *Atmosphere*. These domains may therefore be particularly relevant for understanding how patients orient toward socially salient stimuli in interactive environments.

The domain *Other Persons* captures anomalous interpersonal experiences such as reduced interpersonal resonance, difficulties in immediately grasping others’ intentions or expressions, and forms of social hyperreflexivity^46^. Patients may experience others as distant, opaque, or unpredictable interaction partners, often accompanied by mistrust, social insecurity, and uneasiness with the gaze of others. Against this background, reduced fixation on the NPC’s face may reflect a reluctance to engage in face-directed attention resembling eye contact with a socially ambiguous interaction partner.

A related interpretation concerns the domain *Language*, which refers not only to linguistic deficits but more broadly to alterations in the shared structures of meaning that organize communication and interpersonal understanding. Importantly, this domain includes phenomena such as diminished interpersonal orientation and shifts of attention and context relevance^47^. If the intersubjective framework that normally supports communication is weakened, social signals such as facial expressions, gestures, or gaze direction may become less immediately meaningful. Reduced attention to the NPC’s face could therefore reflect a diminished salience of communicative affordances in socially ambiguous situations.

The domain *Atmosphere* refers to themes such as a diminished sense of reality, altered meaning structures, disrupted familiarity, and a reduced vitality of the surrounding world^48^. Such atmospheres shape how individuals orient within a situation before specific objects or persons become the focus of attention. If a situation is experienced as vague, distant, or unfamiliar, attention may be distributed differently across the environment rather than being drawn to socially salient cues such as faces. In addition, the specific design of the NPC as a robot may evoke an uncanny atmosphere. The negative correlation between atmospheric disturbances and face fixation may therefore indicate that global changes in situational meaning influence patterns of social attention.

Importantly, these associations were stronger than those observed with global symptom severity measured by the PANSS. This finding supports the idea that phenomenological dimensions of experience may map more closely onto subtle behavioral alterations than traditional symptom scales.

Although these preliminary results must be treated with caution in light of the limited sample size, future studies may tend to the possible interpretation that alterations in the lived structure of the social world may manifest behaviorally as subtle changes in how patients detect and engage with socially relevant affordances in interactive virtual environments.

### Ethical considerations

The present study did not reveal marked differences in emotional responses to VR exposure between patients with schizophrenia spectrum disorders and healthy controls. Measures of affect and stress were largely comparable across groups, suggesting that immersive VR environments may generally be tolerated well even in this clinically vulnerable population. Interestingly, although schizophrenia has often been described as involving a private experiential reality alongside the shared social world, patients did not differ from controls in their sense of presence in the virtual environment, suggesting that adaptation to this additional virtual layer of reality is not fundamentally altered.

However, the qualitative observations highlight that immersive environments can interact with ongoing psychotic experiences in complex ways. In one case, a participant used the virtual environment to reinterpret or reenact elements of prior psychotic experiences, while in two other cases the immersive context appeared to influence the meaning and trajectory of ongoing delusional interpretations. Immersive environments may thus become integrated into the sense-making processes through which patients interpret their experiences and surroundings.

Such findings resonate with broader ethical considerations regarding immersive technologies^29^. VR environments can evoke strong perceptual and emotional responses because they engage users in sensorimotor loops that closely resemble real-world perception and action. The psychological and neurobiological consequences of repeated VR exposure remain insufficiently understood, even in healthy populations. Caution may therefore be particularly warranted when applying such technologies in vulnerable groups, including individuals with schizophrenia who may already experience altered perceptions of reality and interpersonal meaning.

Taken together, while the present results suggest that immersive VR exposure is not inherently distressing for patients with schizophrenia spectrum disorders, the qualitative observations underscore the importance of careful monitoring and further systematic research on how immersive environments interact with psychotic experiences.

### Limitations

Several limitations should be considered. The small sample size is a central limitation of the present study. In the absence of prior data to support a conventional a priori power calculation, the study was conducted as an exploratory pilot study with a sample size that was heuristically determined in a conservative way considering the clinically vulnerable population. As revealed by the sensitivity analysis, the final sample limited statistical power, particularly after correction for multiple comparisons.

The clinical sample was diagnostically heterogeneous within the schizophrenia spectrum, which reflects clinical reality but may also have increased interindividual variability and reduced the ability to detect disorder-specific effects.

Moreover, most patients were receiving antipsychotic medication, which may influence perceptual, attentional, and motor processes. Although there was no robust evidence that antipsychotic dose accounted for the behavioral findings after correction for multiple comparison, a moderately negative correlation coefficient with the duration of object interactions warrants consideration of medication effects in future studies.

Furthermore, visual exploration and interaction behavior were manually coded rather than measured with eye-tracking systems, which would allow more precise assessment of gaze behavior.

The self-paced nature of interactions in the VR game may account for differences in the emotional impact of the experiment. However, the total duration did not differ significantly between groups and the reported behavioral variables are largely independent of the total duration.

Because the order of VR conditions was not counterbalanced, possible order effects (e.g., habituation or fatigue) cannot be fully disentangled from condition-specific effects.

The results should therefore be interpreted as preliminary and hypothesis-generating. They require replication in larger, better age-matched samples. Nevertheless, they illustrate the potential of immersive VR paradigms to experimentally investigate alterations of subject–world interaction in schizophrenia.

## Conclusions

Immersive virtual environments can help to explore subtle alterations in how patients with schizophrenia interact with their surroundings and their affordances. Interestingly, some behavioral patterns seemed associated with phenomenological domains of anomalous world experience in this small sample, suggesting that immersive VR paradigms may provide a promising experimental approach to investigate altered subject-world relations in psychosis. Future studies combining immersive environments with larger samples and more precise behavioral measures may further clarify how experiential disturbances relate to observable patterns of environmental exploration and engagement.

## Supplementary information

Supplementary Table 1

Supplementary Table 2

Supplementary Table 3

Supplementary Table 4

## Data Availability

The data that support the findings of this study are available from the corresponding author upon reasonable request and in accordance with potential privacy restrictions.
