# Supplementary material for: Exploring interaction with environmental affordances in schizophrenia spectrum disorders using virtual reality
Source: Schizophrenia (Heidelb). 2026 Jun 25;12(1):64. doi: 10.1038/s41537-026-00774-7 (PMC13401596; doi:10.1038/s41537-026-00774-7)
Supplement: Supplementary file 1 — Supplementary Table 1 [file 41537_2026_774_MOESM1_ESM.docx]

Supplementary Table 1. Video sources and creators.

| **Type** | **Title** | **Hyperlink** | **Creator** |
| --- | --- | --- | --- |
| Urban | Seattle Downtown - City Tour 360 VR - 4K Video. Part 1 - 1 HR | <https://www.youtube.com/watch?v=Zy2ihEV-ooI> | ProArtInc (www.proartwa.com) |
| Urban | Seattle Downtown - City Tour 360 VR - 4K Video. Part 2 - 1 HR | <https://www.youtube.com/watch?v=RDYMD_IZVbo> | ProArtInc (www.proartwa.com) |
| Mountaintop | Virtual Nature Relaxation - VR 360° 5K Video - Chief Mountain, BC, Canada | <https://www.youtube.com/watch?v=__kQAlOJhfQ> | ProArtInc (www.proartwa.com) |
| Riverside | Virtual Nature Relaxation - VR 360° 5K Video - Creek Canyon Trail, BC, Canada | <https://www.youtube.com/watch?v=Kv5ap7VXjys> | ProArtInc (www.proartwa.com) |
| Beach | Malibu Beach - VR 360 - 4K Video - Soothing Surround Beach Sounds - ASMR CaliScapes | <https://www.youtube.com/watch?v=bW9VYhytk-c> | Highway Forty Productions (https://highwayfortyproductions.com/) |
| Forest | 360° Video, Nature Sounds of Forest for Relaxing \| 8K VR video | <https://www.youtube.com/watch?v=wg6eyd6odhU> | NamooFactory (http://namoofactory.com) |
| Savanna | Surrounded by Wild Elephants in 4k 360 | <https://www.youtube.com/watch?v=mlOiXMvMaZo> | Ryan Whitehead (https://www.ryanwhitehead360.com/) |
