# Supplementary material for: Exploring interaction with environmental affordances in schizophrenia spectrum disorders using virtual reality
Source: Schizophrenia (Heidelb). 2026 Jun 25;12(1):64. doi: 10.1038/s41537-026-00774-7 (PMC13401596; doi:10.1038/s41537-026-00774-7)
Supplement: Supplementary file 2 — Supplementary Table 2 [file 41537_2026_774_MOESM2_ESM.docx]

Supplementary Table 2. Action types in the “First Steps” lobby (n, %).

| **Variable** | **SCZ (n=19)** | **HC (n=19)** | **p (uncorrected)** |  |
| --- | --- | --- | --- | --- |
| **Blocks**  - Closely observing  - Knocking over  - Stacking  - Throwing  - Dropping  - Hitting blocks  - Hitting objects with blocks | 4 (21.05%)  1 (5.26%)  4 (21.05%)  5 (26.32%)  6 (31.58%)  2 (10.53%)  8 (42.11%) | 3 (15.89%)  2 (10.53%)  2 (10.53%)  10 (52.63%)  2 (10.53%)  1 (5.26%)  5 (26.32%) | 1.000  1.000  0.660  0.184  0.232  1.000  0.495 |  |
| **Rocket**  - Dropping  - Letting fly  - Throwing  - Hitting objects with rocket | 6 (31.58%)  14 (73.68%)  3 (15.89%)  2 (10.53%) | 5 (26.32%)  16 (84.21%)  5 (26.32%)  1 (5.26%) | 1.000  0.693  0.693  1.000 |  |
| **Paper plane**  - Closely observing  - Letting fly  - Hitting it | 3 (15.89%)  16 (84.21%)  3 (15.89%) | 4 (21.05%)  17 (89.47%)  3 (15.89%) | 1.000  1.000  1.000 |  |
| **Table tennis paddle**  - Dropping  - Throwing  - Hitting the ball  - Hitting other objects | 1 (5.26%)  2 (10.53%)  17 (89.47%)  2 (10.53%) | 0  2 (10.53%)  19 (100%)  4 (21.05%) | 1.000  1.000  0.486  0.660 |  |
| **Punching ball**  - Punching it  - Hitting it with objects | 17 (89.47%)  3 (15.89%) | 18 (94.74%)  3 (15.89%) | 1.000  1.000 |  |
| **RC zeppelin**  - Maneuvering  - Honking  - Hitting it | 18 (94.74%)  12 (63.16%)  1 (5.26%) | 18 (94.74%)  12 (63.16%)  3 (15.89%) | 1.000  1.000  0.604 |  |

Values indicate n (%) of participants who performed each action at least once. Group comparisons were conducted using two-sided Fisher’s exact tests, uncorrected for multiple comparisons.
