# Supplementary material for: Exploring interaction with environmental affordances in schizophrenia spectrum disorders using virtual reality
Source: Schizophrenia (Heidelb). 2026 Jun 25;12(1):64. doi: 10.1038/s41537-026-00774-7 (PMC13401596; doi:10.1038/s41537-026-00774-7)
Supplement: Supplementary file 3 — Supplementary Table 3 [file 41537_2026_774_MOESM3_ESM.docx]

Supplementary Table 3. Correlation Table.

| **Variable** | **PANSS** | **Space and objects** | **Time and events** | **Other persons** | **Language** | **Atmosphere** | **Existential orientation** |
| --- | --- | --- | --- | --- | --- | --- | --- |
| **PANSS** | - | 0.193 | **0.588^†^** | 0.352 | 0.448 | 0.342 | 0.381 |
| **360° video exploration**  **Gaze shifts**  - Urban  - Nature  **Gaze durations**  - Other human beings  - Non-human objects | -0.050  -0.298  -0.219  0.285 | 0.126  0.099  -0.234  0.077 | 0.058  0.150  -0.154  0.037 | 0.330  0.298  -0.371  **0.456^†^** | 0.319  0.235  -0.459  0.399 | 0.343  0.243  -0.030  0.047 | 0.155  0.078  -0.009  -0.080 |
| **Game**  **Object interaction**  - Control difficulties [s]  - Action diversity [n]  - Reaction time [s]  - Interaction duration [s]  **NPC interaction**  - Latency till activation  - Face fixation duration  - Touch interaction | **0.660***  **0.525^†^**  0.399  -0.210  0.044  -0.344  -0.152 | -0.097  0.076  0.004  0.024  0.227  -0.267  -0.227 | 0.115  0.429  0.286  0.079  0.151  -0.388  -0.170 | -0.295  0.225  -0.305  -0.454  -0.285  **-0.669***  -0.412 | -0.184  0.377  -0.237  **-0.509^†^**  -0.236  **-0.677***  -0.408 | -0.008  0.365  -0.032  -0.135  0.052  **-0.615***  -0.474 | 0.053  -0.030  0.205  0.177  0.167  -0.393  -0.330 |

Spearman correlations between PANSS (positive and negative symptom scale) scores, EAWE (examination of anomalous world experience)-derived domains, and behavioral measures of exploration and interaction in virtual reality in the schizophrenia group. * = p < 0.05 (after false discovery rate (FDR) correction); ^†^ = p < 0.05 (not significant after correction).
