# Supplementary material for: Exploring interaction with environmental affordances in schizophrenia spectrum disorders using virtual reality
Source: Schizophrenia (Heidelb). 2026 Jun 25;12(1):64. doi: 10.1038/s41537-026-00774-7 (PMC13401596; doi:10.1038/s41537-026-00774-7)
Supplement: Supplementary file 4 — Supplementary Table 4 [file 41537_2026_774_MOESM4_ESM.docx]

Supplementary Table 4. Correlations between chlorpromazine equivalents and main outcome variables.

| **Variable** | **r (x Chlorpromazine equivalent)** | **P (raw)** | **P (corrected)** |
| --- | --- | --- | --- |
| **PANSS** | 0.477 | 0.061 | 0.366 |
| **360° video exploration**  **Gaze shifts**  - Urban  - Nature  **Gaze durations**  - Other human beings  - Non-human objects | -0.079  -0.187  0.160  -0.013 | 0.771  0.488  0.553  0.964 | 0.925  0.801  0.801  0.964 |
| **Game**  **Object interaction**  - Control difficulties [s]  - Action diversity [n]  - Reaction time [s]  - Interaction duration [s]  **NPC interaction**  - Latency till activation  - Face fixation duration  - Touch interaction | 0.335  0.247  -0.141  -0.553  -0.038  -0.326  -0.284 | 0.205  0.357  0.601  **0.026^†^**  0.888  0.255  0.326 | 0.714  0.714  0.801  0.324  0.964  0.714  0.714 |

Spearman correlations between chlorpromazine equivalents, PANSS (positive and negative symptom scale) scores, and behavioral measures of exploration and interaction in virtual reality in the schizophrenia group. * = p < 0.05 (after false discovery rate (FDR) correction); ^†^ = p < 0.05 (not significant after correction).
